# The Role of Toxic Metals and Metalloids in Nrf2 Signaling

**DOI:** 10.3390/antiox10050630

**Published:** 2021-04-21

**Authors:** Aleksandra Buha, Katarina Baralić, Danijela Djukic-Cosic, Zorica Bulat, Alexey Tinkov, Emiliano Panieri, Luciano Saso

**Affiliations:** 1 Department of Toxicology “Akademik Danilo Soldatović”, Faculty of Pharmacy, University of Belgrade, 11000 Belgrade, Serbia; katarina.baralic@pharmacy.bg.ac.rs (K.B.); danijela.djukic.cosic@pharmacy.bg.ac.rs (D.D.-C.); zorica.bulat@pharmacy.bg.ac.rs (Z.B.); 2 Laboratory of Molecular Dietetics, IM Sechenov First Moscow State Medical University (Sechenov University), 119435 Moscow, Russia; tinkov.a.a@microelements.ru; 3 Laboratory of Ecobiomonitoring and Quality Control, Yaroslavl State University, 150003 Yaroslavl, Russia; 4 Department of Physiology and Pharmacology “Vittorio Erspamer”, Sapienza University P.le Aldo Moro 5, 00185 Rome, Italy; emiliano.panieri@isprambiente.it (E.P.); luciano.saso@uniroma1.it (L.S.)

**Keywords:** metals, metalloid, oxidative stress, Nrf2 pathway, carcinogenesis

## Abstract

Nuclear factor erythroid 2-related factor 2 (Nrf2), an emerging regulator of cellular resistance to oxidants, serves as one of the key defensive factors against a range of pathological processes such as oxidative damage, carcinogenesis, as well as various harmful chemicals, including metals. An increase in human exposure to toxic metals via air, food, and water has been recently observed, which is mainly due to anthropogenic activities. The relationship between environmental exposure to heavy metals, particularly cadmium (Cd), lead (Pb), mercury (Hg), and nickel (Ni), as well as metaloid arsenic (As), and transition metal chromium (Cr), and the development of various human diseases has been extensively investigated. Their ability to induce reactive oxygen species (ROS) production through direct and indirect actions and cause oxidative stress has been documented in various organs. Taking into account that Nrf2 signaling represents an important pathway in maintaining antioxidant balance, recent research indicates that it can play a dual role depending on the specific biological context. On one side, Nrf2 represents a potential crucial protective mechanism in metal-induced toxicity, but on the other hand, it can also be a trigger of metal-induced carcinogenesis under conditions of prolonged exposure and continuous activation. Thus, this review aims to summarize the state-of-the-art knowledge regarding the functional interrelation between the toxic metals and Nrf2 signaling.

## 1. Introduction

### 1.1. Nrf2 Signaling

Nuclear factor erythroid 2-related factor 2 (Nrf2) is a member of the cap ‘‘n’’ collar basic region-leucine zipper transcription factors encoded by the gene nuclear factor erythroid 2-like 2 (NFE2L2) [1]. It is rapidly activated in response to alterations of the redox balance and serves as one of the key defensive factors against a range of pathological processes, including oxidative damage and inflammation [2,3,4,5,6].

**Figure 1:**
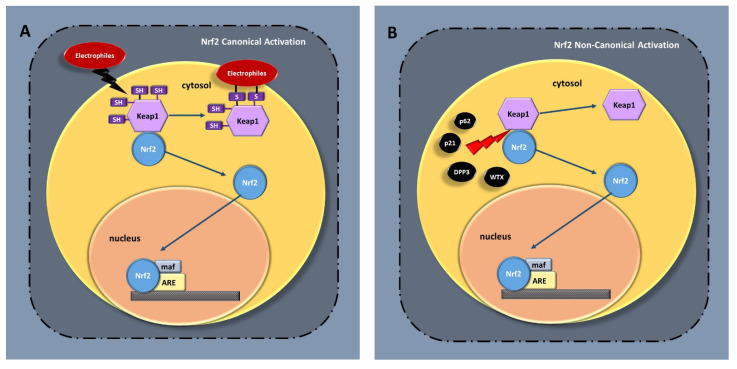

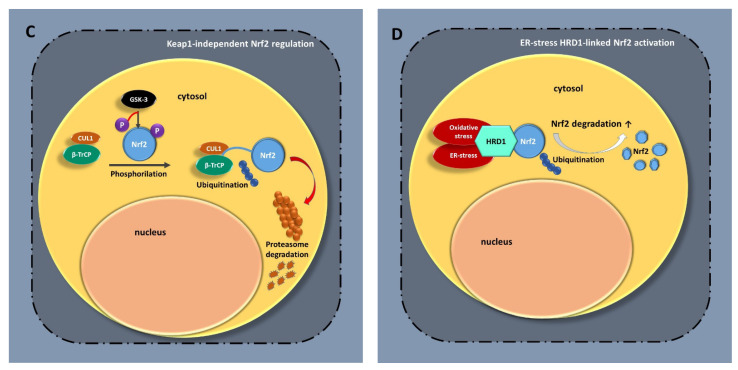
(**A**) Canonical mechanism of Nrf2 activation; (**B**) Non-canonical mechanism of Nrf2 activation; (**C**) Keap1-independent Nrf2 regulation; (**D**) ER-stress HRD-1-linked Nrf2 activation. Nrf2—nuclear factor erythroid 2–related factor 2; Keap1—Kelch-like ECH-associated protein 1; SH—sulfhidrile groups; maf-musculoaponeurotic fibrosarcoma protein; ARE—antioxidant response element; p62—protein p62; p21—protein p21; DPP3—dipeptidyl peptidase III; WTX—wilms tumor gene on X chromosome; β-TrCP—β-transducin repeat-containing protein; CUL1—Skp1–Cul1–Rbx1/Roc1 ubiquitin ligase complex; GSK-3—glycogen synthase kinase-3; HRD-1—E3 ubiquitin ligase; ER—endoplasmic reticulum.

Under physiological conditions, Nrf2 is located in the cytoplasm, bound to its negative regulator, Kelch-like ECH-associated protein 1 (Keap1), and is constantly primed for proteasomal degradation by the Cul3–Rbx1–E3 ubiquitin ligase complex prompted by the interaction between a single Nrf2 protein and a Keap1 homodimer [7]. Following stimulation-induced phosphorylation, Nrf2 is relieved from Keap1 negative regulation by two separate mechanisms [8,9]. The first mechanism, the so-called “canonical pathway”, includes the chemical modification of highly reactive cysteines present in Keap1 BTB and IVR domains, which form protein–protein crosslinks following reaction with electrophiles and lead to the disturbance of the Nrf2 interaction with the Cul3–Keap1 E3 ubiquitin ligase complex, causing decreased Nrf2 proteasomal degradation (Figure 1A) [10,11]. The second mechanism, commonly identified as “non-canonical pathway”, includes a number of different proteins such as p62, p21, dipeptidyl peptidase III (DPP3), PALB2, BRCA1, and Wilms tumor gene on the X chromosome (WTX). These proteins can impair the formation of the Nrf2–Keap1 complex by competing with Keap1 or Nrf2 for their reciprocal binding, ultimately decreasing Nrf2 ubiquitination but increasing its nuclear translocation and activation (Figure 1B) [11]. Interestingly, a number of alternative pathways controlling Nrf2 stability independently from Keap1 function have been described so far. In the first case, the phosphorylation of Nrf2 in its serine-rich Neh6 domain by glycogen synthase kinase-3 (GSK-3) facilitates the recognition of Nrf2 by β-transducin repeat-containing protein (β-TrCP), which is a substrate receptor for the Skp1–Cul1 Rbx1/Roc1 ubiquitin ligase complex that targets Nrf2 for ubiquitination and proteasomal degradation (Figure 1C) [12]. On the other hand, a second mechanism operates through the autophagy–lysosome pathway that normally is involved in the removal of damaged subcellular components including organelles and proteins. Conditions promoting autophagic dysfunction induce the accumulation of the autophagy substrate p62/SQSTM1, which is a multi-domain protein that interacts with a number of molecular partners [13,14]. Among the others, p62 interacts with and sequesters Keap1 into the autophagosomes, thus preventing its recognition by Nrf2 [13,15,16]. Another important pathway at the intersection of oxidative and ER-stress response depends on the E3 ubiquitin ligase HRD1, which is normally involved in the regulation of protein turnover in the ERAD (ER-associated degradation) system. However, it has been shown that under ER stress occurring in cirrothic livers, HRD1 is overexpressed and negatively controls NRF2 stability by promoting its ubiquitination and subsequent degradation [17] (Figure 1D). These mechanisms are believed to finely regulate the degree of Nrf2 pathway activation, preventing the unnecessary induction of the downstream target genes, which instead occurs when Nrf2 escapes from proteasomal degradation and is free to enter into the nucleus.

After the translocation into the nucleus, Nrf2 binds to the Maf proteins [8,9]. Nrf2–Maf heterodimers positively regulate the expression of numerous of detoxification and antioxidant genes that contain a cis-acting element in their promoter region, which is known as the antioxidant response element (ARE). These genes confer protection against oxidative stress and xenobiotics (e.g., NQO1 (NAD(P)H quinone oxidoreductase 1)), promote NADPH regeneration (e.g., G6PD (glucose-6-phosphate dehydrogenase)), and regulate heme and iron metabolism (e.g., HO-1 (heme oxygenase 1)) [3,18,19]. The so-called Nrf2/ARE pathway further initiates the transcription of other target genes coding for proteins responsible for ROS detoxication and increased antioxidant capacity, such as glutathione reductase (GSR), glutathione peroxidase (GSH-Px), catalase (CAT), glutaredoxin (GRX), thioredoxin (TRX), sulfiredoxin (SRX), peroxiredoxin (PRX), and glutamyl cysteine synthetase (GCS) [20,21]. Thus, Nrf2 is not only involved in many diseases wherein oxidative stress plays a causal role (e.g., cardiovascular disease, neuronal degeneration, and cancer) [22] but also functions as a xenobiotic-activated receptor (XAR) to regulate the adaptive response, acting as a powerful protective mechanism for living organisms against exposure to environmental toxicants [1,7,23]. Hence, Nrf2 and *NFE2L2* are known to be affected by many toxic substances, including aflatoxin B1 [24], paraquat [25], phthalates [26], as well as toxic metals and metaloids such as arsenic [27], lead [28], and chromium [29]. Additionally, As, Pb, and Cr have all been shown to dissociate the Nrf2–Keap1 complex. These toxic substances recognize and modify distinct cysteine residues on the Keap1 surface, particularly Cys226 and Cys613 [30]. After this, several sensor systems are formed on the Keap1 molecule, and its function is disrupted, which subsequently leads to Nrf2 derepression/activation [31]. On the other hand, considering that the Nrf2 pathway has been shown to play a protective role against exogenous oxidative damage, many recent studies have attempted to identify a protective agent that might induce specific Nrf2 activation. Thus, various phytochemicals, such as sulforaphane [9,32,33,34], curcumin [20,35], quercetin [3], and mangiferin [36] have been shown to suppress metal-induced toxicity via modulation of the Nrf2-dependent cellular defence mechanisms.

However, in light of its role in carcinogenesis, especially when induced by toxic elements, Nrf2 can be considered a molecule with a dual nature, being both antitumorigenic and protumorigenic. Nrf2 can upregulate anti-apoptotic proteins such as Bcl-2 and Bcl-xL, promoting cell proliferation, thereby contributing to cancer cell survival [37]. Persistent activation and accumulation of Nrf2 due to deregulated signaling and genetic mutations might lead to carcinogenesis, tumor progression, and chemotherapeutic resistance [37,38]. Therefore, gain-of-function mutations in Nrf2 were detected in the skin, esophagus, and larynx carcinomas, while loss-of-function mutations in *KEAP1* were found in lung, bladder, ovary, breast, liver, and stomach carcinomas [37]. Furthermore, apart from the role of the Nrf2–KFAP1 pathway in cancer promotion, its role in inhibition was suggested as well, proposing the possibility of using Nrf2 modulators in cancer therapy [22].

### 1.2. Toxic Metals and Metalloids

Although these elements are naturally present in the Earth’s crust, their content in the environment has increased due to not only natural processes but also anthropogenic activities, such as manufacturing, agriculture, and metal mining [39,40]. Thus, certain metals have become important environmental pollutants, particularly in areas with high anthropogenic pressure [41]. Non-occupationally-exposed individuals can come into contact with these chemicals through a variety of sources, including air, drinking water, food, and tobacco smoke [40,42,43]. Hence, the relationship between environmental exposure to toxic elements, particularly lead (Pb), cadmium (Cd), arsenic (As), and mercury (Hg), but also nickel (Ni), chromium (Cr), cobalt (Co), and antimony (Sb), and human diseases have been repeatedly investigated [44,45,46,47,48,49,50,51]. Some of these elements are known to damage the mental and central nervous activities, lungs, liver, kidneys, endocrine system, as well as other fundamental organs even at low concentrations, causing various diseases [42]. Moreover, several have been shown to be carcinogenic to humans and/or experimental animals, while As, Cd, Cr, and Ni have been classified as group 1 carcinogens by the International Agency for Research on Cancer (IARC) [52,53]. They exhibit their harmful effects, including carcinogenicity, through different mechanisms. These include the induction of oxidative stress by generating free radicals and reducing antioxidant levels, the alteration of DNA structure and expression of miRNA, inhibition of ion channels, ATP-ases and other transporters, the increase in cytoskeleton and cell polarity, or even the impairment of endocytosis and intracellular vesicle recycling [40,54,55].

A thorough knowledge of the molecular mechanisms associated with metal and metalloid toxicity is essential and can contribute to the clinical development of appropriate protective agents against their toxicity. Thus, this review aims to summarize the state-of-the-art knowledge regarding the interrelation between the toxic elements and Nrf2 signaling as one of the important pathways targeted by these substances.

## 2. Determining Toxic Elements Role in Nrf2 Signaling

### 2.1. Cadmium-Associated Changes in Nrf2 Signaling

Cadmium (Cd) is a toxic metal often classified into industrial and environmental pollutants, while diet and tobacco smoking are considered its most important sources for the general population [56,57,58]. Cadmium accumulation may be associated with various toxic effects on the kidney, liver, bone, lung, and testis, [58,59,60,61]. It has been classified as a group 1 carcinogen by the IARC due to its firm linkage to kidney and prostatic cancers [62]; however, its linkage to pancreatic and breast cancer development has also been suggested [44,63,64]. Cadmium is also a well-known endocrine disruptor, its role in thyroid [47], metabolic [46,65], and reproductive disorders [66,67,68] has been suggested and shown in the literature. Mechanisms of Cd toxicity include changes in genes expression and the inhibition of DNA damage repair, interference with autophagy, apoptosis, and alteration of bioelements levels and inhibition of antioxidant defence [56]. The findings from the vast majority of the studies support the notion that Cd exposure stabilizes Nrf2, prevents its degradation, and promotes its nuclear translocation as a protective mechanism [69]. This leads to the dose-dependent increase in the expression of Nrf2-downstream target genes [70], such as NQO1 and HO-1, in organs such as the liver [71,72] and spleen [73], as demonstrated in vivo by rodents studies but also in vitro by experiments on the astrocytoma cell line [74] and Mouse Embryonic Fibroblasts [2]. Lawal and Ellis (2011) speculated that Cd provokes nuclear Nrf2 activation by interacting with a specific G protein-coupled metal-binding receptor in macrophages and inducing phospholipase C. This event causes the release of intracellular Ca^2+^ and diacylglycerol, both of which have the potential to modulate Nrf2 signaling [74]. By using Nrf2 knockout (Nrf2-/-) mouse embryonic fibroblasts cells, it has been demonstrated that the absence of Nrf2 can be connected with a dramatical elevation in ROS production, resulting in increased sensitivity to Cd-induced cell death [75]. In vitro experiments on bovine aortic endothelial cells have provided a link between the upregulation of metallothioneins (MTs), a family of cysteine-rich proteins that can bind Cd, and the Keap1–Nrf2 system upon Cd exposure [76]. This can be considered another proof of the positive impact of the Nrf2 signaling on the defense against Cd toxicity [76], since MTs stimulation serves as an initial response by capturing and incapacitating Cd through thiol groups of the MTs cysteine residues [76]. In contrast, Wu et al. found no relationship between Nrf2 and MTs stimulation in liver after a single dose of CdCl_2_ in mice with enhanced Nrf2 expression [77]. The authors concluded that the positive Nrf2 impact against Cd toxicity, such as the normalization of serum ALT and LDH activities after the Cd exposure and the attenuation of morphological liver alterations, can mostly be linked to the induction of genes involved in the antioxidant defense [77]. However, it was demonstrated that administration of a single CdCl_2_ dose to mice can also contribute to the liver damage by inhibiting Nrf2 and HO-1 and activating inflammatory signaling pathways, NF-κB, NLRP3, and MAPKs [72]. Inhibition of Nrf2 was also found in mice testes accompanied by the decrease of Nrf2 downstream genes, GSH-Px, GCS, HO-1, and NQO1 [32].

As mentioned before, Nrf2 could be regarded as a double-edged sword in the light of carcinogenesis induced by toxic metals, including Cd. In the first stage of Cd-induced carcinogenesis, the antioxidant activity of Nrf2 decreases ROS and shelters cells from the malignant transformation. However, in the second stage of Cd-induced carcinogenesis, continuous Nrf2 activation contributes to the acquired apoptosis resistance, providing a favorable environment to cell survival and tumorigenesis [78,79].

Park and Seo (2011) have demonstrated that DNA damage occurs in the Cd treated-Nrf2 lacking the colon cancer RKO cell line, which is followed by the increased intracellular ROS generation and induction of micronuclei (MN), which is a hallmark of carcinogenicity in cadmium-exposed Nrf2 deficient cells. The authors concluded that Nrf2 has important roles in the suppression of the carcinogenicity of cadmium in terms of protection against oxidative stress-induced DNA damage [80]. However, by performing in vitro experiments on human bronchial epithelial cells (BEAS-2BR cell line), Wang et al. have suggested the ability of Cd to suppress autophagy, resulting in the accumulation of autophagosomes and increased p62 protein. This is followed by a positive feedback mechanism, wherein p62 upregulates Nrf2, which also further upregulates p62. Eventually, constitutive Nrf2 activation increases downstream anti-apoptotic proteins, Bcl-2 and Bcl-xl, resulting in apoptosis resistance and possible carcinogenicity [78].

Some of the most important studies investigating the effects of Cd on Nrf2 signaling using in vitro and in vivo models are summarized in Table 1.

### 2.2. Lead-Associated Changes in Nrf2 Signaling

Lead (Pb) is an omnipresent toxic metal widely used in industry and found not only in environmental air, dust, foods, and drinking water but also in various products such as cosmetics, toys, paints, shot bullets, fishing weights, and lead-acid batteries [82]. Lead is recognized as an environmental pollutant threatening human health, even in low doses, and causing severe neurological, hematological, gastrointestinal, immunological, cardiovascular, and reproductive disorders [82,83,84]. The results of both in vitro and in vivo studies have shown that exposure to Pb can be linked to a decreased antioxidant enzymes activity and expression, while authors have suggested the potential relationship with Nrf2 signaling [28,85]. In bovine granulosa cells, by disrupting Nrf2/NF-κB interaction, exposure to Pb induced oxidative stress, attenuated cell proliferation, and altered cell cycle progression, ultimately inducing apoptosis. Significant reduction in the Keap1 mRNA levels was observed, while Nrf2 showed a significant decrease with concomitant downregulation of both SOD and CAT levels [85]. Albarakati et al. also confirmed that Pb treatment caused a decrement in antioxidant enzyme activity and expression (SOD, CAT, GSH-Px, and GR) and led to the high malondialdehyde (MDA) levels in rat kidneys. In the same study, Pb exposure downregulated *Nfe2l2* and HO-1 mRNA expression and amplified the levels of inflammatory markers (TNF-α, IL-1β, and NO) in renal tissue, which were upregulated [28]. Downregulated gene expression of Nrf2, NQO-1, and HO-1 could be seen in the rat testis as well resulting in oxidative damage, inflammation, and cell death [82]. Moreover, inhibition of Nrf2 and HO-1 activation, coupled with the decreased nuclear translocation of Nrf2 in mice brain, could be connected with oxidative stress, inflammation, and apoptosis of neurons in hippocampus tissue extracted from mice after 4-week Pb treatment [86]. Furthermore, the Nrf2/HO-1 signaling pathway was highlighted as a cellular self-defense mechanism against Pb-induced oxidative stress in SH-SY5Y cells as well, confirming its important role in neuroprotection [83]. Moreover, Ye et al. demonstrated the rapid elevation of Nrf2 nuclear accumulation and Nrf2–ARE binding activities after the exposure of the same cell line to Pb. The authors also identified a link between Nrf2 and gene expression, concluding that Pb could induce mRNA transcription of HO-1, glutathione S-transferase omega 2 (GSTa1), and NQO1, as well as the protein expression of HO-1 and g-glutamylcysteine synthetase (g-GCS), which are all controlled by Nrf2 [87]. Another organ in which AMPK/Nrf2/p62 signaling was suggested to have a defensive role from oxidative stress, inflammation, and apoptosis were lungs, as demonstrated in an experiment in which rats were exposed to Pbvia drinking water [88]. Moreover, Wang et al. have shown a significant increase in the Nrf2 expression in testes after the administration of different Pb doses to rats via drinking water from weaning to 6 months of age. The authors observed nuclear translocation of Nrf2, along with the dose-dependent decrease in GST and GSH, suggesting that the multidrug resistance protein 1 (Mrp1), which was also upregulated upon Pb exposure, might play important roles in lead detoxification by Nrf2 [89]. The authors speculated that overactivation of nuclear factor kappa B (NF-κB) by free radical overproduction increased the level of Keap1, leading to Nrf2 impairment and a decrease in its antioxidant effects [90].

Some of the most important studies investigating the role of this metal on Nrf2 signaling are summarized in Table 2.

### 2.3. Arsenic-Associated Changes in Nrf2 Signaling

Inorganic As (III) is a ubiquitous environmental contaminant that occurs in both food and air, while intake through water consumption presents a severe worldwide health issue [91]. Chronic As exposure has been found to provoke various detrimental effects and disrupt normal cellular homeostasis, particularly associated with respiratory dysfunctions [36], and linked to chronic obstructive pulmonary disease (COPD), asthma, and pulmonary fibrosis [50,91,92]. Based on sufficient evidence for carcinogenicity, inorganic As compounds are classified by IARC as “human carcinogens”, due to the confirmed relationship between chronic exposure to As and skin, lung, bladder, liver, kidney, and pancreatic cancers [62].

Findings regarding the role of Nrf2 signaling in As toxicity can be considered conflicting. While the Nrf2 signaling pathway plays a significant role in the antioxidation process and is considered of great importance for the prevention and treatment of arsenic poisoning [38], hyperactivation of the Nrf2 pathway, which occurs in the case of chronic As exposure, contributes to cell proliferation and carcinogenicity [92]. Aono et al. were the first to propose the involvement of Nrf2 signaling in As toxicity by demonstrating Nrf2 activation after 16h exposure of osteoblasts to 800 µM of As (V). This event was followed by the activation of target genes encoding HO-1, as well as other oxidative stress-inducible proteins that play important roles in the protective mechanisms under critical conditions for cell survival, such as Peroxiredoxin I (Prx I) and A170 [93,94]. Furthermore, He et al. concluded that Nrf2 is required for induction of the detoxification gene, NQO1, in mouse hepa1c1c7 cells after As exposure [95]. The positive role of Nrf2 signaling was similarly noted after the exposure of mice to As via drinking water, in a concentration that produces a blood arsenic level comparable to that of humans living in areas where arsenic exposure is endemic [96]. Accordingly, another in vivo investigation has hinted at a protective role of Nrf2 against oxidative stress, demonstrating upregulation of the transcriptional expression of genes encoding antioxidant enzymes, SOD1 and GSH-Px1, in rat serum and liver after the As exposure in various concentrations, both via food and drinking water [97]. Increase in SOD2 and HO-1 synthesis linked to the upregulated expression of Nrf2 protein was also noted in mice lungs after As treatment for 3 months [36].

However, after the exposure of the bronchial epithelial cell line BEAS-2B to different As concentrations, Bi et al. have found the relationship between Nrf2 and hypoxia-inducible factor 1α (HIF1α), including their mutual transcriptional regulation, identifying Nrf2 activation as an initiating signal for As-induced HIF1α activation. This aspect is of particular relevance, since both Nrf2 and HIF1α can participate in the transcriptional regulation of the key genes important for malignant transformation and the generation of cancer stem-like cells [98]. Schmidin et al. have found that chronic As exposure enhances the invasive and migratory capacity of immortalized lung epithelial cells via Nrf2-dependent upregulation of SRY-box 9 (SOX9), which is a transcription factor linked to cell proliferation, epithelial–mesenchymal transition, and metastasis [92]. Accordingly, the authors concluded that hyperactivation of the Nrf2 gene via the knockout of Keap1 contributes to cell proliferation, while inhibition of Nrf2 or direct knockdown of SOX9 affects the cells’ ability to proliferate, migrate, and invade [92]. Pi et al. have concluded that constitutive Nrf2 activation may be involved in arsenic skin carcinogenesis, demonstrating generalized apoptotic resistance after exposing human HaCaT keratinocytes to 100 nM As for 28 days. Another finding of this study potentially substantiating the involvement of Nrf2 signaling in As-induced carcinogenesis is that biomarkers for malignant transformation, MMP-9, and various cytokeratins, were potentially regulated by Nrf2 [27]. Wu et al. have also demonstrated the ability of As to induce malignant transformation of HaCaT, resulting in an enhanced p62-NRF2 feedback loop [99]. Finally, Shah et al. have shown that chronic arsenic exposure elevates p62 protein levels in the epidermis of mice skin. The authors concluded that the modulation of the Nrf2 pathway was responsible for the arsenic-induced p62 expression in keratinocytes and suggested that targeting p62 may assist in preventing arsenic-induced skin cancer [100].

Furthermore, a recent study in rats revealed the possible role of Nrf2 signaling in the neuroendocrine disruption caused by arsenic trioxide. Namely, gestational exposure to arsenic trioxide initiated hypothyroidism/thyrotoxicity in both dams and fetuses indicating fetal thyroid-cerebrum axis disruption partly relying on the suppression of Nrf2 mRNA expression and PPARγ as revealed in the fetal cerebrum in both maternal treated groups [101].

Some of the most important studies investigating the role of As in Nrf2 signaling are presented in Table 3.

### 2.4. Mercury-Associated Changes in Nrf2 Signaling

Mercury (Hg) represents a ubiquitous environmental toxic metal that could lead to severe toxic effects in a variety of organs usually at a low level [35]. It readily infiltrates the blood–brain barrier and disturbs the central nervous system, especially when present in its organic form, methylmercury (MeHg). It is markedly bioaccumulated by aquatic organisms and, thus, can be found at the highest concentrations in top predators of the aquatic food chain [103].

In vivo studies have confirmed the relationship between exposure to inorganic Hg, Nrf2, and oxidative stress in various organs, e.g., heart [10] and liver [35]. For example, Baiyun et al. (2018) have demonstrated that as a result of 56-days HgCl_2_ treatment, Nrf2 accumulation in the nucleus was significantly decreased in the cardiac tissue of rats, which was connected with HgCl_2_-induced ROS production in the heart [10]. Similarly, in another study, even after 3 days of exposure, HgCl_2_ treatment activated the Nrf2–ARE pathway in rats [35]. The results of this study have also demonstrated that Nrf2 expression was upregulated in a concentration-dependent manner after HgCl_2_ exposure, indicating that HgCl_2_ could promote the dissociation of Nrf2 from its negative regulator Keap 1, resulting in the induction of cytoprotective proteins. The authors speculated that the chemical interaction of Hg^2+^ with Keap 1 protein thiol, followed by the disruption of the thiol groups via oxidative modifications, might contribute to the upregulation of Nrf2 expression [35]. However, after 2 weeks of exposure of rats to HgCl_2_ through drinking water, increased cell death in rat liver could be attributed to inefficient ROS scavenging due to a failure in Nrf2 activation [104].

It is also evident that cells respond to organic mercury, particularly MeHg, through the activation of Nrf2 associated with S-mercuration of Keap1, and that the Keap1/Nrf2 pathway protects against MeHg toxicity [103]. It is important to note that Keap1 is a cysteine-rich protein possessing 27 and 25 cysteine residues in humans and mice, respectively. Hence, it is possible that MeHg modifies Keap1 through S-mercuration of specific cysteines and activates the Nrf2-regulated gene expression of glutamate–cysteine ligase, HO-1, and multidrug resistance protein [103]. Evidence also suggests thatf Keap1-independent signal pathways can play a significant role, which might provide an additional contribute to MeHg-induced Nrf2 activation and cytoprotective responses against MeHg exposure. These include Akt phosphorylation (Akt/GSK-3b/Fyn-mediated Nrf2 activation pathway), activation of the PTEN/Akt/CREB pathway, MAPK-induced autophagy, and increased p62 expression [105]. Thus, it is not surprising that both in vivo and in vitro studies have shown the relationship between MeHg exposure and Nrf2. For example, 22 days of oral exposure of Nrf2-deficient mice to MeHg led not only to the decrease in the body weight but also to the induction of hind-limb flaccidity, while wild-type mice did not show any abnormalities [106]. The linkage between MeHg and Nrf2 has been demonstrated on rat astrocytes as well [20]. However, interestingly, MeHg induced cytotoxicity by promoting the Nrf2/ARE signaling pathway.

Selected studies investigating the role of Hg in Nrf2 signaling are presented in Table 4.

### 2.5. Nickel-Associated Changes in Nrf2 Signaling

Nickel has been considered a hazardous metal as a result of its capability to induce cytotoxicity and carcinogenicity due to environmentally persistent exposure [3,108]. It has been demonstrated that after entering the body, Ni induces tissue damage in various organs, mainly by ROS generation and increased DNA methylation [3]. This toxic metal has been classified by IARC as a group 1 carcinogen due to the confirmed linkage to the increased risks of nasal and lung cancer [109].

Accordingly, the results of an in vivo study have demonstrated that exposure to Ni can be associated with DNA methylation and inflammation connected to the Nrf2/HO-1 and p38/STAT1/NF-κB pathways in the livers of Ni treated mice [3]. Since in this study Ni led to the global hypermethylation but caused DNA hypomethylation of the Nrf2 promoter CpG islands, the authors suggested that this could account for the inhibition of the Nrf2 expression and, consequently, the suppression of Nrf2 activity and its downstream target genes [3]. Accordingly, Kim et al. identified 10 genes (CAV1, FOSL2, MICA, PIM2, RUNX1, SLC7A6, APLP1, CLSPN, PCAF, and PRAME) as potential molecular candidates for Nrf2-related cellular protection against Ni exposure, since they are involved in a variety of molecular processes including oxidative stress response, necrosis, DNA repair, and cell survival. These authors also suggested that Nrf2 can be considered an important factor with a protective role in the suppression of mutagenicity and carcinogenicity induced by Ni exposure [108]. By showing that Ni not only increased whole-cell Nrf2 levels and nuclear translocation in human monocytic cells but also amplified lipopolysaccharide (LPS)-induction of Nrf2, Lewis et al. concluded that Ni also affected cytokine secretion through Nrf2 pathway modulation [110]. In data-mining in silico study, a link between the Ni exposure, ER-stress, and Nrf2 signaling has been found [111]. The authors stated that this link is particularly important for insoluble Ni compounds (i.e., Ni_3_S_2_, Ni(CO)_4_), since they are uptaken by the cell via phagocytosis. Subsequently, after Ni^2+^ dissociation, this metal is aggregated near the nucleus, and it might directly interact with DNA [111]. Selected studies investigating the role of Ni in Nrf2 signaling are presented in Table 5.

### 2.6. Chromium-Associated Changes in Nrf2 Signaling

Widespread industrial usage together with unrestrained environmental release has made Cr both an occupational hazard and an aquatic contaminant [2]. It not only leads to respiratory hazards such as perforation of nasal mucosa and asthma but is also classified as a group 1 carcinogen by IARC due to its linkage to lung, nasal cavity, and paranasal sinus cancer development [112,113].

Both in vitro and in vivo studies have demonstrated that Cr exposure is connected with elevated ROS production and apoptosis, identifying the Nrf2 pathway as a vitally important mechanism for protecting cells subjected to oxidative stress [2,9,29,83,114,115,116]. Using mouse hepa1c1c7 cells, it was demonstrated that after the Cr exposure, activation of the Nrf2 pathway and induction of cytoprotective genes HO-1 and NQO1 occurs [2]. The resulting inhibition of Nrf2 and its accumulation into the nucleus was followed by the nuclear translocation and deubiquitination of Keap1, which was further recycled, forming a transcriptional signaling loop [2]. Nrf2 signaling has been found to be an important mechanism in controlling ROS-induced cytotoxicity of liver cells after the exposure of rats to a single dose of K_2_Cr_2_O_7_ by activating antioxidant enzymes and protecting the hepatocytes [115]. In other studies, an even lower single dose of K_2_Cr_2_O_7_ was found to decrease the expression of P-AMPK/AMPK and Nrf2 in rat heart, followed by oxidative stress, apoptosis, and release of inflammatory mediators [29], while the same dose administered longer not only caused a significant decrease in Nrf2 but also affected Sirt1, Pgc-1α, HO-1, and NQO1 expression in rat lungs [8]. Furthermore, by using the zebrafish model, Shaw et al. (2019) demonstrated that even environmentally relevant Cr concentration may initiate the increase in Nrf2 on both the transcriptional and translational levels, as well as enhance its nuclear translocation [116].

Some of the in vitro and in vivo studies investigating the role of Cr in Nrf2 signaling are presented in Table 6.

## 3. Conclusions

Nrf2 signaling represents an important pathway in maintaining antioxidant balance, while recent research has highlighted its interrelation with metal- and metalloids- induced toxicity. However, regardless of the type of study (in vitro vs. in vivo), conflicting data have been obtained concerning the toxic elements influence on Nrf2 signaling. While some results indicate that toxic metals/metalloids lead to the increase in Nrf2 activation by Keap1-depended mechanisms, conferring protection against their toxicity, others led to the opposite conclusion that the Nrf2–ARE defense pathway is inhibited under the influence of such exposure. However, whether the final outcome of the Nrf2 activation process could be regarded as positive, or negative was found to be largely dependent on the length of the exposure. In this respect, the majority of the studies characterized by shorter exposure time have shown that the Nrf2 signaling pathway plays a significant role in the defense against toxic elements. However, in the case of chronic exposure, especially regarding As and Cd, increased p62 expression and consequent Nrf2 hyperactivation contributed to cell proliferation and carcinogenicity. Thus, it is important to bear in mind that Nrf2 signaling can act as a double-edged sword, decreasing ROS and sheltering cells from the malignant transformation in the first stage, while leading to the suppression of autophagy and apoptosis sensitivity, providing a favorable environment for cell survival and tumorigenesis in the case of continuous activation. Thus, the identification, validation, and optimization of new Nrf2 activators are needed for the development of effective prophylaxis, or therapy that can enhance the Nrf2-dependent adaptive system and protect humans from not only toxic metals/metalloids but also other environmental insults. Furthermore, further investigation of Nrf2 signaling role in the metal- and metalloid-induced carcinogenesis, but also in various other toxic effects of metals and metalloids, such as endocrine disruption, may have significance in creating prophylactic or therapeutic strategy against various illnesses.

## Figures and Tables

**Table 1:** Summary of studies analyzing the effects of Cd on Nrf2 signaling.

Type	Cell Culture/ Species	Treatment Doses	Duration	Effects on Nrf2 Signaling	Ref.
**In vitro**	Nrf2 knockout (Nrf2-/-) mouse embryonic fibroblasts (MEF) cells	2, 5, 10, 50, and 100 μM CdCl_2_	5 h	Increase in the ROS production and increased sensitivity to Cd-induced cell death in Nrf2 knockout (Nrf2-/-) MEF cells.	[75]
	rat proximal tubular (rPT) cells	2.5 µM Cd	12 h	Oxidative stress via Nrf2 antioxidant pathway. Enhanced Nrf2 nuclear translocation. Downmodulation of Keap1. Activated Nrf2 target genes, including detoxifying enzymes (NQO1 and HO-1). Autophagosome accumulation.	[69]
	astrocytoma cell line 1321N1	5 and 10 µM Cd	24 h	Increased levels of NQO1 and HO-1 mRNA. Increased nuclear accumulation of Nrf2. Connection between phospholipase C activation and Nrf2 signaling.	[74]
	RKO human colon carcinoma cell line	0, 5, 10, 20, 40, 80, 160 and 320 μM CdCl_2_	24 h	DNA damage and increased intracellular ROS generation in Nrf2 lacking RKO cells. Induction of micronuclei (MN)—hallmark of carcinogenicity in Cd-exposed Nrf2 deficient cells. Nrf2—important role in suppression of Cd-induced carcinogenicity.	[80]
	bovine aortic endothelial cells	0.5, 1, 2, 5 µM CdCl_2_	24 h	Modification of cysteine residues in Keap1 and Nrf2 activation. Up-regulation of metallothionein. Participation of Keap1–Nrf2 system in the modulation of metallothionein-1/2 expression.	[76]
	BEAS-2BR lung cells	5 or 20 µM CdCl_2_	24 h	Autophagy deficiency, accumulation of autophagosomes, and increased p62. Nrf2-p62 positive feedback mechanism. Constitutive Nrf2 activation increases anti-apoptotic proteins, Bcl-2 and Bcl-xl. Apoptosis resistance.	[78]
**In vivo**	zebrafish	0, 11, and 110 μg·L^−1^ CdCl_2_	24 h	Dose-dependent induction of Nrf2-regulated antioxidant genes. Increased glutathione S-transferase pi, glutamate–cysteine ligase catalytic subunit, HO-1 and peroxiredoxin 1 mRNA.	[70]
	mice	4 mg/kg b.w. CdCl_2_ i.p.	single dose	Activated NF-κB, NLRP3, and MAPKs signaling pathways in liver. Inhibition of Nrf2, HO-1, and activation of NF-κB, NLRP3, and MAPKs contribute to liver injury.	[72]
	mice	3.5 mg/kg b.w. CdCl_2_ i.p.	single dose	Nrf2 activation prevents Cd-induced oxidative stress and liver injury through induction of genes involved in antioxidant defense rather than genes that scavenge Cd (metallothioneins).	[77]
	mice	6.5 mg/kg b.w. CdCl_2_ i.p.	7 days	Impaired expression of Nrf2 gene in testes.	[81]
	mice	2.3 mg/kg b.w. CdCl_2_ i.p.	10 days	Reduced mRNA and protein expression of mouse testicular Nrf2. Decreased expression of Nrf2 downstream genes, GSH-Px, glutamyl cysteine synthetase (GCS), HO-1, NQO1.	[32]
	rats	20 mg/L CdCl_2_ Drinking water	8 weeks	Increased Nrf2 nuclear translocation. Elevated expression of Nrf2-downstream targets in rat liver. Cd-elevated protein levels of hepatic antioxidant enzymes.	[71]
	rats	20 mg/L CdCl_2_ Drinking water	8 weeks	Increased Nuclear translocation of Nrf2 in spleen. Induction of apoptosis and inhibition of autophagy.	[73]

Abbreviations: body weight (b.w.), intraperironeal (i.p.).

**Table 2:** Summary of studies analyzing the effects of Pb on Nrf2 signaling.

Type of Study	Cell Culture/ Species	Treatment Concentration/Dose	Duration	Effects on Nrf2 Signaling	Ref.
**In vitro**	bovine granulosa cells	1, 2, 3, 5, and 10 μg/mL	2 h	Oxidative stress that attenuates cell proliferation and alters cell cycle progression. Apoptosis through disrupted Nrf2/NF-κB interaction. Decrease in Nrf2. Concomitant downregulation of both SOD and CAT.	[85]
	SH-SY5Y cells	1, 5, 25 or 125 μM Pb (CH_3_COO)_2_	24 h	Nrf2/HO-1 signaling pathway as cellular self-defense mechanism protects against Pb-induced oxidative stress.	[83]
	SH-SY5Y cells	125 μM Pb (CH_3_COO)_2_	3, 6, 12 and 24 h	Rapid increase in Nrf2 nuclear accumulation. Nrf2–ARE binding activities in a ROS-dependent manner. Nrf2 regulated induction of mRNA transcription of HO-1, GSTa1, and NQO1, as well as the protein expression of HO-1 and g-GCS.	[87]
**In vivo**	rats	20 mg/kg b.w., Pb (CH_3_COO)_2_ i.p.	7 days	Downmodulation of antioxidant enzyme activity and expression in renal tissue (SOD, CAT, GSH-Px). High MDA levels. Downregulation of Nfe212 and Homx1 mRNA expression. Increased inflammatory markers (TNF-α, IL-1β and NO). Upregulated synthesis of apoptotic related proteins. Downregulated anti-apoptotic protein expression.	[28]
	rats	20 mg/kg b.w. Pb (CH_3_COO)_2_ i.p.	7 days	Pb (CH_3_COO)_2_ deactivated Nrf2 and HO-1 in the testicular tissue. Overactivation of nuclear factor kappa B (NF-κB) by free radical overproduction, increased the level of Keap1, leading to Nrf2 impairment and decrease in its antioxidant effect.	[90]
	rats	50 mg/kg b.w. Pb (CH_3_COO)_2_ Oral gavage	4 weeks	Downregulated gene expression of testicular Nrf2, NQO-1, and HO-1. Oxidative damage, inflammation, and cell death.	[82]
	mice	250 mg/L Pb (CH_3_COO)_2_ Drinking water	4 weeks	Apoptosis of neurons in hippocampus tissue. Oxidative stress, inflammation, and apoptosis by inhibiting the activations of Nrf2 HO-1 in rat brain. Decreased nuclear translocation of Nrf2 and the protein expressions of HO-1 and NQO1.	[81]
	rats	2500 ppm Pb (CH_3_COO)_2_ Drinking water	5 weeks	AMPK/Nrf2/p62 signaling protects the lung from oxidative stress, inflammation, and apoptosis.	[88]
	rats	0, 0.3, and 0.9 g/L Pb (CH_3_COO)_2_ Drinking water	6 months	Significant increases in the expressions of Mrp1 and Nrf2 in rat testes at both administered dose levels. Increased nuclear translocation of Nrf2. Dose-dependent decrease in GST and GSH. Mrp1—important roles in lead detoxification by Nrf2.	[89]

Abbreviations: body weight (b.w.), intraperironeal (i.p.).

**Table 3:** Summary of the studies analyzing the effects of As on Nrf2 signaling.

Type of Study	Cell Culture/ Species	Treatment Doses	Duration	Effects on Nrf2 Signaling	Ref.
	mouse hepa1c1c7 cells	2.5 and 10 µM NaAsO_2_	5 h	Nrf2 is required for induction of detoxification gene, NQO1. As extended the t1⁄2 of Nrf2 by inhibiting the Keap1–Cul3-dependent ubiquitination and proteasomal Nrf2 turnover. As did not disrupt the Nrf2–Keap1–Cul3 association in the cytoplasm, but it induced Nrf2 dissociation from Keap1 and Cul3 and dimerization of Nrf2 with a Maf protein (Maf G/Maf K) in nucleus.	[98]
**In vitro**	bronchial epithelial cell line BEAS-2B	0, 0.25, 0.5, 1, 2, 4 µM As^3+^(inorganic)	8 h	Binding of Nrf2 and/or HIF1α on the genome. Amplified Nrf2 enrichment peaks in intergenic region, promoter and gene body. Mutual transcriptional regulation between Nrf2 and HIF1α. Nrf2 activation is an initiating signal for As-induced HIF1α activation.	[98]
	L-02 cells	25 μM NaAsO_2_	24 h	Decreased Nrf2 and its downstream genes expression.	[38]
	non-small cell lung cancer (NSCLC)	0.5 μM NaAsO_2_	3 months	Chronic As exposure enhances the invasive and migratory capacity of immortalized lung epithelial cells via Nrf2-dependent upregulation of SRY-box 9 (SOX9), transcription factor linked with cell proliferation, epithelial-mesenchymal transition, and metastasis. Hyperactivation of Nrf2 gene via knockout of Keap1 contributes to cell proliferation.	[92]
	human HaCaT keratinocytes	4 and 8 μM NaAsO_2_	28 weeks	As induces p62 expression to form a positive feedback loop with Nrf2.	[100]
	human HaCaT keratinocytes	100 nM NaAsO_2_	28 weeks	Increased intracellular glutathione and elevated expression of Nrf2 and its target genes. Generalized apoptotic resistance. Diminished Nrf2-mediated antioxidant response induced by acute exposure to high doses of arsenite. Biomarkers for malignant transformation, MMP-9, and cytokeratins, are potentially regulated by Nrf2. Constitutive Nrf2 activation may be involved in arsenic skin carcinogenesis.	[27]
	human keratinocytes (HaCaT)	100 nM or 200 nM NaAsO_2_	4 h	Silencing NRF2 abrogated the increase in mRNA and protein levels of p62 and malignant phenotypes induced by arsenite	[99]
	MC3T3-E1 osteoblasts	800 µM NaAsO_2_	16 h	Nrf2 activation. Transcriptional activation of target genes encoding HO-1, Prx I, and A170.	[93]
**In vivo**	Nrf2-WT and Nrf2-KO mice	5 mg NaAsO_2_ Oral gavage/ 20 ppm NaAsO_2_ Drinking water	single dose/ 6 weeks	Increased basal transcript levels of GSTa1 and significantly lower GST mu 1 (Gstm1) in liver of Nrf2-KO mice compared to Nrf2-WT control.	[102]
	mice	10 mg/kg b.w. NaAsO_2_ Drinking water	3 months	An upregulated expression of Nrf2 protein in mice lungs. Nrf2 has a pivotal role to maintain the endogenous redox balance. Induced synthesis of antioxidants SOD2 and HO-1.	[36]
	mice (Nrf2+/+ and Nrf2−/−)	5 ppm NaAsO_2_ Drinking water	4 months	Decrease in the bone volume in mice lacking Nrf2. Lack of Nrf2 increases As-induced ROS levels and phosphorylation of p38.	[96]
	rats	5 mg/kg b.w. NaAsO_2_ Oral gavage	28 days	Increased levels of ROS, 8-hydroxydeoxyguanosine (8-OHdG) and lipid peroxidation in kidney. Decreased levels of enzymatic and non-enzymatic antioxidants. Increase in apoptotic markers, DNA damage, TUNEL-positive cells, and dark staining of ICAM-1 in renal tissue with decreased PI3K/Akt/Nrf2 gene expression.	[33]
	rats	100 mg/L Drinking water /25, 50, 100 mg/kg as food	90 days	Oxidative stress in rat liver related to the PKCδ-Nrf2-ARE signaling pathway. Nrf2 was associated with upregulation of the transcriptional expression of SOD1 and GSH-Px1 in each arsenic poisoning group.	[97]

Abbreviation: body weight (b.w.).

**Table 4:** Summary of studies analyzing the effects of Hg on Nrf2 signaling.

Type of Study	Cell Culture/ Species	Treatment Doses	Duration	Effects on Nrf2 Signaling	Ref.
**In vitro**	rat astrocytes	5 μM MeHg	6h	Cytotoxicity by promoting the Nrf2/ARE signaling pathway.	[20]
**In vivo**	yellow croaker *Pseudosciaena crocea*	0,32 and 64 μg/L HgCl_2_	96h	A coordinated transcriptional regulation of antioxidant genes, by Nrf2 in liver. A negative relationship between the mRNA levels of Nrf2 and Keap1 indicated that Keap1 may play an important role in switching off the Nrf2 response.	[107]
	homozygous (–/–) Nrf2-deficient mice (C57BL/6J) and wild-type (+/+) mice	1 mg/kg MeHg Oral gavage	22 days	MeHg in Nrf2-deficient mice—induction of hind-limb flaccidity. The body weight decrease of Nrf2-deficient mice.	[106]
	rats	80 mg/L HgCl_2_ Drinking water	56 days	Decreased Nrf2 accumulation in the nucleus in the cardiac tissue. Decreased GSH level and GSH/GSSG ratio, increased MDA concentration in the heart.	[10]
	rats	0.6, 1.2, and 2.4 mg/kg HgCl_2_ i.p.	3 days	Nrf 2 activation in liver. Upregulation HO-1, and γ-GCS heavy subunit expression.	[35]
	rats	80 mg/L HgCl_2_ Drinking water	2 weeks	Increased hepatocyte death attributed to insufficient ROS removal because of a failure in Nrf2 activation.	[104]

Abbreviation: intraperironeal (i.p.).

**Table 5:** Summary of studies analyzing the effects of Ni on Nrf2 signaling.

Type of Study	Cell Culture/ Species	Treatment Doses and Duration	Duration	Effects on Nrf2 Signaling	Ref.
**In vitro**	human monocytic cells	10–30 mM Ni (II)	6–72 h	Increased whole-cell Nrf2 levels and nuclear translocation of Nrf2. Affected cytokine secretion through Nrf2 pathway modulation.	[110]
	RKO (ATCC CRL-2577), human colon cancer cells	20 μM Ni(CH_3_CO_2_)_2_·x H_2_O	12 or 24 h	Nrf2 gene silencing exacerbated Ni-induced oxidative stress and DNA damage.	[108]
**In vivo**	mice	20 mg/kg/b.w. NiSO_4_(H_2_O)_6_ i.p.	20 days	DNA methylation and liver inflammation associated with the Nrf2/HO-1 and p38/STAT1/NF-κB pathways.	[3]

Abbreviation: intraperironeal (i.p.).

**Table 6:** Summary of studies analysing the effects of Cr on Nrf2 signaling.

Type of Study	Cell Culture/ Species	Treatment Doses	Duration	Effects on Nrf2 Signaling	Ref.
**in vitro**	mouse hepa1c1c7 cells	2, 5, 10, 50, and 100 M Cr(VI)	5 h	Elevated ROS production and apoptosis. Protection by Nrf2 correlates with the induction of cytoprotective genes HO-1 and NQO1. Inhibition of ubiquitination of Nrf2 and accumulation of Nrf2 into the nucleus. Nuclear translocation and deubiquitination of Keap1. Transcriptional signaling loop: activation of Nrf2 by Cr, transcription of ARE-driven genes, and reduction of ROS production.	[2]
**in vivo**	zebrafish	38.16 μg/mL K2CrO4	1, 7, 15, 30, or 60 days	Increased Nrf2 in liver both at transcriptional and translational level. Nrf2 translocation into the nucleus. Oxidative stress resulting in lipid peroxidation and extensive changes in tissue ultrastructure.	[116]
	grass carp *Ctenopharyngodon idellus*	5.3 and 10.63 mg/L	15, 30 or 45 days	Alteration in the gene expression of Nrf2 and Mt2 in gills. Development of oxidative stress.	[114]
	rats	4 mg/kg b.w. K_2_Cr_2_O_7_ i.p.	35 days	Significantly decrease in Sirt1, Pgc-1α, Nrf2, HO-1, and NQO1 in rat lungs.	[8]
	rats	17 mg/kg b.w. K_2_Cr_2_O_7_ i.p.	single dose	Nrf2 signaling—important mechanism in controlling liver cells susceptibility to ROS-induced cytotoxicty. Nrf2 increase activates antioxidant enzymes.	[115]
	rats	4 mg/kg K_2_Cr_2_O_7_ i.p.	single dose	Decreased expression of P-AMPK/AMPK and Nrf2. Oxidative stress, apoptosis, and the release of inflammatory mediators in the rat heart.	[29]
	rats	4 mg/kg b.w. K_2_Cr_2_O_7_ i.p.	35 days	Nrf2 pathway—critical protective role against oxidative stress in heart.	[9]

Abbreviations: body weight (b.w.), intraperironeal (i.p.).

## References

[1] He F., Ru X., Wen T. (2020). NRF2, a Transcription Factor for Stress Response and Beyond. Int. J. Mol. Sci..

[2] He X., Lin G.X., Chen M.G., Zhang J.X., Ma Q. (2007). Protection against Chromium (VI)-Induced Oxidative Stress and Apoptosis by Nrf2. Recruiting Nrf2 into the Nucleus and Disrupting the Nuclear Nrf2/Keap1 Association. Toxicol. Sci..

[3] Liu C.-M., Ma J.-Q., Xie W.-R., Liu S.-S., Feng Z.-J., Zheng G.-H., Wang A.-M. (2015). Quercetin protects mouse liver against nickel-induced DNA methylation and inflammation associated with the Nrf2/HO-1 and p38/STAT1/NF-κB pathway. Food Chem. Toxicol..

[4] Vomund S., Schäfer A., Parnham M., Brüne B., von Knethen A. (2017). Nrf2, the Master Regulator of Anti-Oxidative Responses. Int. J. Mol. Sci..

[5] Tonelli C., Chio I.I.C., Tuveson D.A. (2018). Transcriptional Regulation by Nrf2. Antioxid. Redox Signal..

[6] Bellezza I., Giambanco I., Minelli A., Donato R. (2018). Nrf2-Keap1 signaling in oxidative and reductive stress. Biochim. Biophys. Acta-Mol. Cell Res..

[7] Osburn W., Kensler T. (2008). Nrf2 signaling: An adaptive response pathway for protection against environmental toxic insults. Mutat. Res. Mutat. Res..

[8] Han B., Li S., Lv Y., Yang D., Li J., Yang Q., Wu P., Lv Z., Zhang Z. (2019). Dietary melatonin attenuates chromium-induced lung injury via activating the Sirt1/Pgc-1α/Nrf2 pathway. Food Funct..

[9] Yang D., Han B., Baiyun R., Lv Z., Wang X., Li S., Lv Y., Xue J., Liu Y., Zhang Z. (2020). Sulforaphane Attenuates Hexavalent Chromium-Induced Cardiotoxicity via Activation of the Sesn2/AMPK/Nrf2 Signaling Pathway. Metallomics.

[10] Baiyun R., Li S., Liu B., Lu J., Lv Y., Xu J., Wu J., Li J., Lv Z., Zhang Z. (2018). Luteolin-mediated PI3K/AKT/Nrf2 signaling pathway ameliorates inorganic mercury-induced cardiac injury. Ecotoxicol. Environ. Saf..

[11] Silva-Islas C.A., Maldonado P.D. (2018). Canonical and non-canonical mechanisms of Nrf2 activation. Pharmacol. Res..

[12] Ahmed S.M.U., Luo L., Namani A., Wang X.J., Tang X. (2017). Nrf2 signaling pathway: Pivotal roles in inflammation. Biochim. Biophys. Acta-Mol. Basis Dis..

[13] Komatsu M., Kurokawa H., Waguri S., Taguchi K., Kobayashi A., Ichimura Y., Sou Y.S., Ueno I., Sakamoto A., Tong K.I. (2010). The selective autophagy substrate p62 activates the stress responsive transcription factor Nrf2 through inactivation of Keap1. Nat. Cell Biol..

[14] Lau A., Wang X.-J., Zhao F., Villeneuve N.F., Wu T., Jiang T., Sun Z., White E., Zhang D.D. (2010). A Noncanonical Mechanism of Nrf2 Activation by Autophagy Deficiency: Direct Interaction between Keap1 and p62. Mol. Cell. Biol..

[15] Lau A., Zheng Y., Tao S., Wang H., Whitman S.A., White E., Zhang D.D. (2013). Arsenic Inhibits Autophagic Flux, Activating the Nrf2-Keap1 Pathway in a p62-Dependent Manner. Mol. Cell. Biol..

[16] Copple I.M., Lister A., Obeng A.D., Kitteringham N.R., Jenkins R.E., Layfield R., Foster B.J., Goldring C.E., Park B.K. (2010). Physical and functional interaction of sequestosome 1 with Keap1 regulates the Keap1-Nrf2 cell defense pathway. J. Biol. Chem..

[17] Wu T., Zhao F., Gao B., Tan C., Yagishita N., Nakajima T., Wong P.K., Chapman E., Fang D., Zhang D.D. (2014). Hrd1 suppresses Nrf2-mediated cellular protection during liver cirrhosis. Genes Dev..

[18] Hennig P., Garstkiewicz M., Grossi S., Di Filippo M., French L., Beer H.-D. (2018). The Crosstalk between Nrf2 and Inflammasomes. Int. J. Mol. Sci..

[19] Sinha D., Biswas J., Bishayee A. (2013). Nrf2-mediated redox signaling in arsenic carcinogenesis: A review. Arch. Toxicol..

[20] Yang B., Yin C., Zhou Y., Wang Q., Jiang Y., Bai Y., Qian H., Xing G., Wang S., Li F. (2019). Curcumin protects against methylmercury-induced cytotoxicity in primary rat astrocytes by activating the Nrf2/ARE pathway independently of PKCδ. Toxicology.

[21] Baird L., Yamamoto M. (2020). The Molecular Mechanisms Regulating the KEAP1-NRF2 Pathway. Mol. Cell. Biol..

[22] Panieri E., Buha A., Telkoparan-akillilar P., Cevik D., Kouretas D., Veskoukis A., Skaperda Z., Tsatsakis A., Wallace D., Suzen S. (2020). Potential applications of NRF2 modulators in cancer therapy. Antioxidants.

[23] Ma Q. (2013). Role of Nrf2 in Oxidative Stress and Toxicity. Annu. Rev. Pharmacol. Toxicol..

[24] Pauletto M., Tolosi R., Giantin M., Guerra G., Barbarossa A., Zaghini A., Dacasto M. (2020). Insights into Aflatoxin B1 Toxicity in Cattle: An In Vitro Whole-Transcriptomic Approach. Toxins.

[25] Yao J., Zhang J., Tai W., Deng S., Li T., Wu W., Pu L., Fan D., Lei W., Zhang T. (2019). High-Dose Paraquat Induces Human Bronchial 16HBE Cell Death and Aggravates Acute Lung Intoxication in Mice by Regulating Keap1/p65/Nrf2 Signal Pathway. Inflammation.

[26] Wang X., Han B., Wu P., Li S., Lv Y., Lu J., Yang Q., Li J., Zhu Y., Zhang Z. (2020). Dibutyl phthalate induces allergic airway inflammation in rats via inhibition of the Nrf2/TSLP/JAK1 pathway. Environ. Pollut..

[27] Pi J., Diwan B.A., Sun Y., Liu J., Qu W., He Y., Styblo M., Waalkes M.P. (2008). Arsenic-induced malignant transformation of human keratinocytes: Involvement of Nrf2. Free Radic. Biol. Med..

[28] Albarakati A.J.A., Baty R.S., Aljoudi A.M., Habotta O.A., Elmahallawy E.K., Kassab R.B., Abdel Moneim A.E. (2020). Luteolin protects against lead acetate-induced nephrotoxicity through antioxidant, anti-inflammatory, anti-apoptotic, and Nrf2/HO-1 signaling pathways. Mol. Biol. Rep..

[29] Li J., Zheng X., Ma X., Xu X., Du Y., Lv Q., Li X., Wu Y., Sun H., Yu L. (2019). Melatonin protects against chromium(VI)-induced cardiac injury via activating the AMPK/Nrf2 pathway. J. Inorg. Biochem..

[30] Suzuki T., Motohashi H., Yamamoto M. (2013). Toward clinical application of the Keap1-Nrf2 pathway. Trends Pharmacol. Sci..

[31] Baird L., Dinkova-Kostova A.T. (2011). The cytoprotective role of the Keap1-Nrf2 pathway. Arch. Toxicol..

[32] Yang S.-H., Long M., Yu L.-H., Li L., Li P., Zhang Y., Guo Y., Gao F., Liu M.-D., He J.-B. (2016). Sulforaphane Prevents Testicular Damage in Kunming Mice Exposed to Cadmium via Activation of Nrf2/ARE Signaling Pathways. Int. J. Mol. Sci..

[33] Thangapandiyan S., Ramesh M., Miltonprabu S., Hema T., Jothi G.B., Nandhini V. (2019). Sulforaphane potentially attenuates arsenic-induced nephrotoxicity via the PI3K/Akt/Nrf2 pathway in albino Wistar rats. Environ. Sci. Pollut. Res..

[34] Yang S.-H., Yu L.-H., Li L., Guo Y., Zhang Y., Long M., Li P., He J.-B. (2018). Protective Mechanism of Sulforaphane on Cadmium-Induced Sertoli Cell Injury in Mice Testis via Nrf2/ARE Signaling Pathway. Molecules.

[35] Liu W., Xu Z., Li H., Guo M., Yang T., Feng S., Xu B., Deng Y. (2017). Protective effects of curcumin against mercury-induced hepatic injuries in rats, involvement of oxidative stress antagonism, and Nrf2-ARE pathway activation. Hum. Exp. Toxicol..

[36] Mahalanobish S., Saha S., Dutta S., Sil P.C. (2019). Mangiferin alleviates arsenic induced oxidative lung injury via upregulation of the Nrf2-HO1 axis. Food Chem. Toxicol..

[37] Kim J., Keum Y.-S. (2016). NRF2, a Key Regulator of Antioxidants with Two Faces towards Cancer. Oxid. Med. Cell. Longev..

[38] Xu M., Niu Q., Hu Y., Feng G., Wang H., Li S. (2019). Proanthocyanidins Antagonize Arsenic-Induced Oxidative Damage and Promote Arsenic Methylation through Activation of the Nrf2 Signaling Pathway. Oxid. Med. Cell. Longev..

[39] Rahman Z., Singh V.P. (2019). The relative impact of toxic heavy metals (THMs) (arsenic (As), cadmium (Cd), chromium (Cr)(VI), mercury (Hg), and lead (Pb)) on the total environment: An overview. Environ. Monit. Assess..

[40] Wallace D.R., Taalab Y.M., Heinze S., Tariba Lovaković B., Pizent A., Renieri E., Tsatsakis A., Farooqi A.A., Javorac D., Andjelkovic M. (2020). Toxic-Metal-Induced Alteration in miRNA Expression Profile as a Proposed Mechanism for Disease Development. Cells.

[41] Martí-Cid R., Llobet J.M., Castell V., Domingo J.L. (2008). Dietary Intake of Arsenic, Cadmium, Mercury, and Lead by the Population of Catalonia, Spain. Biol. Trace Elem. Res..

[42] Vardhan K.H., Kumar P.S., Panda R.C. (2019). A review on heavy metal pollution, toxicity and remedial measures: Current trends and future perspectives. J. Mol. Liq..

[43] Andjelkovic M., Djordjevic A.B., Antonijevic E., Antonijevic B., Stanic M., Kotur-Stevuljevic J., Spasojevic-Kalimanovska V., Jovanovic M., Boricic N., Wallace D. (2019). Toxic effect of acute cadmium and lead exposure in rat blood, liver, and kidney. Int. J. Environ. Res. Public Health.

[44] Djordjevic V.R., Wallace D.R., Schweitzer A., Boricic N., Knezevic D., Matic S., Grubor N., Kerkez M., Radenkovic D., Bulat Z. (2019). Environmental cadmium exposure and pancreatic cancer: Evidence from case control, animal and in vitro studies. Environ. Int..

[45] Wallace D.R., Buha Djordjevic A. (2020). Heavy metal and pesticide exposure: A mixture of potential toxicity and carcinogenicity. Curr. Opin. Toxicol..

[46] Buha A., Dukić-Ćosić D., Ćurčić M., Bulat Z., Antonijević B., Moulis J.M., Goumenou M., Wallace D. (2020). Emerging links between cadmium exposure and insulin resistance: Human, animal, and cell study data. Toxics.

[47] Buha A., Matovic V., Antonijevic B., Bulat Z., Curcic M., Renieri E.A., Tsatsakis A.M., Schweitzer A., Wallace D. (2018). Overview of cadmium thyroid disrupting effects and mechanisms. Int. J. Mol. Sci..

[48] Das K.K., Reddy R.C., Bagoji I.B., Das S., Bagali S., Mullur L., Khodnapur J.P., Biradar M.S. (2018). Primary concept of nickel toxicity-An overview. J. Basic Clin. Physiol. Pharmacol..

[49] Bjørklund G., Mutter J., Aaseth J. (2017). Metal chelators and neurotoxicity: Lead, mercury, and arsenic. Arch. Toxicol..

[50] Nurchi V.M., Djordjevic A.B., Crisponi G., Alexander J., Bjørklund G., Aaseth J. (2020). Arsenic toxicity: Molecular targets and therapeutic agents. Biomolecules.

[51] Bjørklund G., Crisponi G., Nurchi V.M., Buha Djordjevic A., Aaseth J. (2019). A Review on Coordination Properties of Thiol-Containing Chelating Agents Towards Mercury, Cadmium, and Lead. Molecules.

[52] Hartwig A. (1998). Carcinogenicity of metal compounds: Possible role of DNA repair inhibition. Toxicol. Lett..

[53] Kim H.S., Kim Y.J., Seo Y.R. (2015). An Overview of Carcinogenic Heavy Metal: Molecular Toxicity Mechanism and Prevention. J. Cancer Prev..

[54] Kim J.-J., Kim Y.-S., Kumar V. (2019). Heavy metal toxicity: An update of chelating therapeutic strategies. J. Trace Elem. Med. Biol..

[55] Sabolić I. (2006). Common Mechanisms in Nephropathy Induced by Toxic Metals. Nephron Physiol..

[56] Đukić-Ćosić D., Baralić K., Javorac D., Djordjevic A.B., Bulat Z. (2020). An overview of molecular mechanisms in cadmium toxicity. Curr. Opin. Toxicol..

[57] Repić A., Bulat P., Antonijević B., Antunović M., Džudović J., Buha A., Bulat Z. (2020). The influence of smoking habits on cadmium and lead blood levels in the Serbian adult people. Environ. Sci. Pollut. Res..

[58] Satarug S. (2018). Dietary Cadmium Intake and Its Effects on Kidneys. Toxics.

[59] Yang S.-H., Li P., Yu L.-H., Li L., Long M., Liu M.-D., He J.-B. (2019). Sulforaphane Protect Against Cadmium-Induced Oxidative Damage in mouse Leydigs cells by Activating Nrf2/ARE Signaling Pathway. Int. J. Mol. Sci..

[60] Buha A., Jugdaohsingh R., Matovic V., Bulat Z., Antonijevic B., Kerns J.G., Goodship A., Hart A., Powell J.J. (2019). Bone mineral health is sensitively related to environmental cadmium exposure- experimental and human data. Environ. Res..

[61] Genchi G., Sinicropi M.S., Lauria G., Carocci A., Catalano A. (2020). The effects of cadmium toxicity. Int. J. Environ. Res. Public Health.

[62] IARC Working Group on the Evaluation of Carcinogenic Risks to Humans (2012). Arsenic, metals, fibres, and dusts. 2012;100(PT C):11. IARC Monogr. Eval. Carcinog. Risks Humans.

[63] Wallace D., Spandidos D., Tsatsakis A., Schweitzer A., Djordjevic V., Djordjevic A. (2019). Potential interaction of cadmium chloride with pancreatic mitochondria: Implications for pancreatic cancer. Int. J. Mol. Med..

[64] Van Maele-Fabry G., Lombaert N., Lison D. (2016). Dietary exposure to cadmium and risk of breast cancer in postmenopausal women: A systematic review and meta-analysis. Environ. Int..

[65] Bimonte V.M., Besharat Z.M., Antonioni A., Cella V., Lenzi A., Ferretti E., Migliaccio S. (2021). The endocrine disruptor cadmium: A new player in the pathophysiology of metabolic diseases. J. Endocrinol. Investig..

[66] de Angelis C., Galdiero M., Pivonello C., Salzano C., Gianfrilli D., Piscitelli P., Lenzi A., Colao A., Pivonello R. (2017). The environment and male reproduction: The effect of cadmium exposure on reproductive functions and its implication in fertility. Reprod. Toxicol..

[67] Tariba Lovaković B. (2020). Cadmium, arsenic, and lead: Elements affecting male reproductive health. Curr. Opin. Toxicol..

[68] Silva N., Peiris-John R., Wickremasinghe R., Senanayake H., Sathiakumar N. (2012). Cadmium a metalloestrogen: Are we convinced?. J. Appl. Toxicol..

[69] Zhang C., Lin J., Ge J., Wang L.-L., Li N., Sun X.-T., Cao H.-B., Li J.-L. (2017). Selenium triggers Nrf2-mediated protection against cadmium-induced chicken hepatocyte autophagy and apoptosis. Toxicol. Vitr..

[70] Wang L., Gallagher E.P. (2013). Role of Nrf2 antioxidant defense in mitigating cadmium-induced oxidative stress in the olfactory system of zebrafish. Toxicol. Appl. Pharmacol..

[71] Gong Z.-G., Wang X.-Y., Wang J.-H., Fan R.-F., Wang L. (2019). Trehalose prevents cadmium-induced hepatotoxicity by blocking Nrf2 pathway, restoring autophagy and inhibiting apoptosis. J. Inorg. Biochem..

[72] Liu C., Zhu Y., Lu Z., Guo W., Tumen B., He Y., Chen C., Hu S., Xu K., Wang Y. (2019). Cadmium Induces Acute Liver Injury by Inhibiting Nrf2 and the Role of NF-κB, NLRP3, and MAPKs Signaling Pathway. Int. J. Environ. Res. Public Health.

[73] Qu K.-C., Wang Z.-Y., Tang K.-K., Zhu Y.-S., Fan R.-F. (2019). Trehalose suppresses cadmium-activated Nrf2 signaling pathway to protect against spleen injury. Ecotoxicol. Environ. Saf..

[74] Lawal A.O., Ellis E.M. (2011). Nrf2-mediated adaptive response to cadmium-induced toxicity involves protein kinase C delta in human 1321N1 astrocytoma cells. Environ. Toxicol. Pharmacol..

[75] He X., Chen M.G., Ma Q. (2008). Activation of Nrf2 in Defense against Cadmium-Induced Oxidative Stress. Chem. Res. Toxicol..

[76] Shinkai Y., Kimura T., Itagaki A., Yamamoto C., Taguchi K., Yamamoto M., Kumagai Y., Kaji T. (2016). Partial contribution of the Keap1–Nrf2 system to cadmium-mediated metallothionein expression in vascular endothelial cells. Toxicol. Appl. Pharmacol..

[77] Wu K.C., Liu J.J., Klaassen C.D. (2012). Nrf2 activation prevents cadmium-induced acute liver injury. Toxicol. Appl. Pharmacol..

[78] Wang Y., Mandal A.K., Son Y.-O., Pratheeshkumar P., Wise J.T.F., Wang L., Zhang Z., Shi X., Chen Z. (2018). Roles of ROS, Nrf2, and autophagy in cadmium-carcinogenesis and its prevention by sulforaphane. Toxicol. Appl. Pharmacol..

[79] Xu J., Wise J.T.F., Wang L., Schumann K., Zhang Z., Shi X. (2017). Dual Roles of Oxidative Stress in Metal Carcinogenesis. J. Environ. Pathol. Toxicol. Oncol..

[80] Park J.Y., Seo Y.R. (2011). The protective role of Nrf2 in cadmium-induced DNA damage. Mol. Cell. Toxicol..

[81] Almeer R., Soliman D., Kassab R., AlBasher G., Alarifi S., Alkahtani S., Ali D., Metwally D., Abdel Moneim A. (2018). Royal Jelly Abrogates Cadmium-Induced Oxidative Challenge in Mouse Testes: Involvement of the Nrf2 Pathway. Int. J. Mol. Sci..

[82] Alotaibi M.F., Al-Joufi F., Abou Seif H.S., Alzoghaibi M.A., Djouhri L., Ahmeda A.F., Mahmoud A.M. (2020). Umbelliferone Inhibits Spermatogenic Defects and Testicular Injury in Lead-Intoxicated Rats by Suppressing Oxidative Stress and Inflammation, and Improving Nrf2/HO-1 Signaling. Drug Des. Devel. Ther..

[83] Yang L., Li X., Jiang A., Li X., Chang W., Chen J., Ye F. (2020). Metformin alleviates lead-induced mitochondrial fragmentation via AMPK/Nrf2 activation in SH-SY5Y cells. Redox Biol..

[84] Javorac D., Signević A.B., Signelković M., Tatović S., Baralić K., Antonijević E., Kotur-Stevuljević J., Signukić-Äosić D., Antonijević B., Bulat Z. (2020). Redox and essential metal status in the brain of Wistar rats acutely exposed to a cadmium and lead mixture. Arh. Hig. Rada Toksikol..

[85] Aglan H.S., Gebremedhn S., Salilew-Wondim D., Neuhof C., Tholen E., Holker M., Schellander K., Tesfaye D. (2020). Regulation of Nrf2 and NF-κB during lead toxicity in bovine granulosa cells. Cell Tissue Res..

[86] Liu C.-M., Tian Z.-K., Zhang Y.-J., Ming Q.-L., Ma J.-Q., Ji L.-P. (2020). Effects of Gastrodin against Lead-Induced Brain Injury in Mice Associated with the Wnt/Nrf2 Pathway. Nutrients.

[87] Ye F., Li X., Li L., Lyu L., Yuan J., Chen J. (2015). The role of Nrf2 in protection against Pb-induced oxidative stress and apoptosis in SH-SY5Y cells. Food Chem. Toxicol..

[88] Lu J., Jiang H., Liu B., Baiyun R., Li S., Lv Y., Li D., Qiao S., Tan X., Zhang Z. (2018). Grape seed procyanidin extract protects against Pb-induced lung toxicity by activating the AMPK/Nrf2/p62 signaling axis. Food Chem. Toxicol..

[89] Wang Y., Fang J., Huang S., Chen L., Fan G., Wang C. (2013). The chronic effects of low lead level on the expressions of Nrf2 and Mrp1 of the testes in the rats. Environ. Toxicol. Pharmacol..

[90] AL-Megrin W.A., Alomar S., Alkhuriji A.F., Metwally D.M., Mohamed S.K., Kassab R.B., Abdel Moneim A.E., El-Khadragy M.F. (2020). Luteolin protects against testicular injury induced by lead acetate by activating the Nrf2/ HO -1 pathway. IUBMB Life.

[91] Lv X., Li Y., Xiao Q., Li D. (2019). Daphnetin activates the Nrf2-dependent antioxidant response to prevent arsenic-induced oxidative insult in human lung epithelial cells. Chem. Biol. Interact..

[92] Schmidlin C.J., Zeng T., Liu P., Wei Y., Dodson M., Chapman E., Zhang D.D. (2020). Chronic arsenic exposure enhances metastatic potential via NRF2-mediated upregulation of SOX9. Toxicol. Appl. Pharmacol..

[93] Aono J., Yanagawa T., Itoh K., Li B., Yoshida H., Kumagai Y., Yamamoto M., Ishii T. (2003). Activation of Nrf2 and accumulation of ubiquitinated A170 by arsenic in osteoblasts. Biochem. Biophys. Res. Commun..

[94] Nakaso K., Kitayama M., Ishii T., Bannai S., Yanagawa T., Kimura K., Nakashima K., Ohama E., Yamada K. (1999). Effects of kainate-mediated excitotoxicity on the expression of rat counterparts of A170 and MSP23 stress proteins in the brain. Mol. Brain Res..

[95] He X., Chen M.G., Lin G.X., Ma Q. (2006). Arsenic Induces NAD(P)H-quinone Oxidoreductase I by Disrupting the Nrf2·Keap1·Cul3 Complex and Recruiting Nrf2·Maf to the Antioxidant Response Element Enhancer. J. Biol. Chem..

[96] Liu Z., Hou Y., Li L., Yang Y., Jia J., Hong Z., Li T., Xu Y., Fu J., Sun Y. (2019). Nrf2 deficiency aggravates the increase in osteoclastogenesis and bone loss induced by inorganic arsenic. Toxicol. Appl. Pharmacol..

[97] Hu Y., Yu C., Yao M., Wang L., Liang B., Zhang B., Huang X., Zhang A. (2018). The PKCδ-Nrf2-ARE signalling pathway may be involved in oxidative stress in arsenic-induced liver damage in rats. Environ. Toxicol. Pharmacol..

[98] Bi Z., Zhang Q., Fu Y., Wadgaonkar P., Zhang W., Almutairy B., Xu L., Rice M., Qiu Y., Thakur C. (2020). Nrf2 and HIF1α converge to arsenic-induced metabolic reprogramming and the formation of the cancer stem-like cells. Theranostics.

[99] Wu X., Sun R., Wang H., Yang B., Wang F., Xu H., Chen S., Zhao R., Pi J., Xu Y. (2019). Enhanced p62-NRF2 Feedback Loop due to Impaired Autophagic Flux Contributes to Arsenic-Induced Malignant Transformation of Human Keratinocytes. Oxid. Med. Cell. Longev..

[100] Shah P., Trinh E., Qiang L., Xie L., Hu W.-Y., Prins G., Pi J., He Y.-Y. (2017). Arsenic Induces p62 Expression to Form a Positive Feedback Loop with Nrf2 in Human Epidermal Keratinocytes: Implications for Preventing Arsenic-Induced Skin Cancer. Molecules.

[101] Ahmed R.G., El-Gareib A.W. (2019). Gestational Arsenic Trioxide Exposure Acts as a Developing Neuroendocrine-Disruptor by Downregulating Nrf2/PPARγ and Upregulating Caspase-3/NF-ĸB/Cox2/BAX/iNOS/ROS. Dose-Response.

[102] Wang H., Zhu J., Li L., Li Y., Lv H., Xu Y., Sun G., Pi J. (2017). Effects of Nrf2 deficiency on arsenic metabolism in mice. Toxicol. Appl. Pharmacol..

[103] Kumagai Y., Kanda H., Shinkai Y., Toyama T. (2013). The Role of the Keap1/Nrf2 Pathway in the Cellular Response to Methylmercury. Oxid. Med. Cell. Longev..

[104] Zhang H., Tan X., Yang D., Lu J., Liu B., Baiyun R., Zhang Z. (2017). Dietary luteolin attenuates chronic liver injury induced by mercuric chloride via the Nrf2/NF-κB/P53 signaling pathway in rats. Oncotarget.

[105] Unoki T., Akiyama M., Kumagai Y., Gonçalves F.M., Farina M., da Rocha J.B.T., Aschner M. (2018). Molecular Pathways Associated With Methylmercury-Induced Nrf2 Modulation. Front. Genet..

[106] Toyama T., Shinkai Y., Yasutake A., Uchida K., Yamamoto M., Kumagai Y. (2011). Isothiocyanates Reduce Mercury Accumulation via an Nrf2-Dependent Mechanism during Exposure of Mice to Methylmercury. Environ. Health Perspect..

[107] Zeng L., Zheng J.-L., Wang Y.-H., Xu M.-Y., Zhu A.-Y., Wu C.-W. (2016). The role of Nrf2/Keap1 signaling in inorganic mercury induced oxidative stress in the liver of large yellow croaker Pseudosciaena crocea. Ecotoxicol. Environ. Saf..

[108] Kim H.L., Seo Y.R. (2012). Molecular and genomic approach for understanding the gene-environment interaction between Nrf2 deficiency and carcinogenic nickel-induced DNA damage. Oncol. Rep..

[109] International Agency for Research on Cancer (IARC) (2018). Nickel Nickel Compounds.

[110] Lewis J.B., Messer R.L., McCloud V.V., Lockwood P.E., Hsu S.D., Wataha J.C. (2006). Ni(II) activates the Nrf2 signaling pathway in human monocytic cells. Biomaterials.

[111] Jiménez-Vidal L., Espitia-Pérez P., Torres-Ávila J., Ricardo-Caldera D., Salcedo-Arteaga S., Galeano-Páez C., Pastor-Sierra K., Espitia-Pérez L. (2019). Nuclear factor erythroid 2–related factor 2 and its relationship with cellular response in nickel exposure: A systems biology analysis. BMC Pharmacol. Toxicol..

[112] Shaw P., Sen A., Mondal P., Dey Bhowmik A., Rath J., Chattopadhyay A. (2020). Shinorine ameliorates chromium induced toxicity in zebrafish hepatocytes through the facultative activation of Nrf2-Keap1-ARE pathway. Aquat. Toxicol..

[113] International Agency for Research on Cancer (IARC) (2018). Chromium (VI) Compounds.

[114] Jindal R., Handa K. (2019). Hexavalent chromium-induced toxic effects on the antioxidant levels, histopathological alterations and expression of Nrf2 and MT2 genes in the branchial tissue of Ctenopharyngodon idellus. Chemosphere.

[115] Kalayarasan S., Sriram N., Sureshkumar A., Sudhandiran G. (2008). Chromium (VI)-induced oxidative stress and apoptosis is reduced by garlic and its derivative *S-* allylcysteine through the activation of Nrf2 in the hepatocytes of Wistar rats. J. Appl. Toxicol..

[116] Shaw P., Mondal P., Bandyopadhyay A., Chattopadhyay A. (2019). Environmentally relevant concentration of chromium activates Nrf2 and alters transcription of related XME genes in liver of zebrafish. Chemosphere.

